# Porewater compositions of Portland cement with and without silica fume calculated using the fine-tuned CASH+NK solid solution model

**DOI:** 10.1617/s11527-022-02045-0

**Published:** 2022-09-24

**Authors:** George Dan Miron, Dmitrii A. Kulik, Barbara Lothenbach

**Affiliations:** 1 Laboratory for Waste Management LES, Paul Scherrer Institut, 5232 Villigen, Switzerland; 2 Concrete and Asphalt Laboratory, Empa, 8600 Dübendorf, Switzerland

**Keywords:** Calcium silicate hydrates, C–S–H, Portland cement, Silica fume, Thermodynamic modelling, Alkali uptake

## Abstract

The CASH+ sublattice solid solution model of C–S–H aims to predict the composition of C–S–H and its ability to take up alkalis. It was originally developed for dilute systems with high water–solid ratios, and thus in this paper further optimized and benchmarked against measured pore solution compositions of hydrated Portland cement (PC) and PC blended with silica fume (SF) at realistic water-binder ratios. To get an improved agreement with the pore solution data, the stability of two CASH+ model endmembers, TCKh and TCNh, has been fine-tuned with standard Gibbs energy corrections of + 7.0 and + 5.0 kJ·mol^−1^, respectively (at 1 bar, 25 °C). The agreement was maintained with the experiments used to originally parameterize the CASH+ model for the uptake of K and Na in dilute systems. The K and Na concentrations predicted using the fine-tuned CASH+NK model are in a good agreement with the measured values for PC and PC + SF system at different water to binder ratios, silica fume additions, and at temperatures up to 80 °C.

**Supplementary Information:**

The online version contains supplementary material available at 10.1617/s11527-022-02045-0.

## Introduction

The presence of alkali metals in cementitious materials is of great importance as the alkali concentration plays a key role for the chemical and mechanical properties of cementitious materials. The alkali metal concentration in the pore solution strongly affects the pH of the solution, which in turn influences the aqueous speciation and the concentrations of the other dissolved components. Thus, the alkali concentrations influence the stability of cement hydrates [1], stability of the steel passivation layer [2], contaminants mobility and sorption [3, 4], reactivity of supplementary cementitious materials [5, 6], and influence degradation processes such as the alkali-silica-reaction (ASR) [7, 8].

K and Na are the main alkali metals present in cements while the amount of other alkali metals such as Li, Rb, Cs is low. Alkali metals are present in Portland cements partially in the form of easily soluble sulfates, and partially incorporated in the clinker phases and are released when the clinker reacts [9–11]. Upon hydration, they distribute between the pore solution and the solid hydration products, where they are binding mainly in the abundant C–S–H phase [12–14]. Thus, the alkali binding by C–S–H controls the distribution of alkali metals between solids and aqueous solution, and hence the pH values in the pore solution in Portland cements and blended cements [15]. To be able to correctly predict pH values and alkali metal concentrations in the pore solution of cements the thermodynamic model describing C–S–H phases needs to correctly account for the uptake of Na and K.

**Fig. 1:**
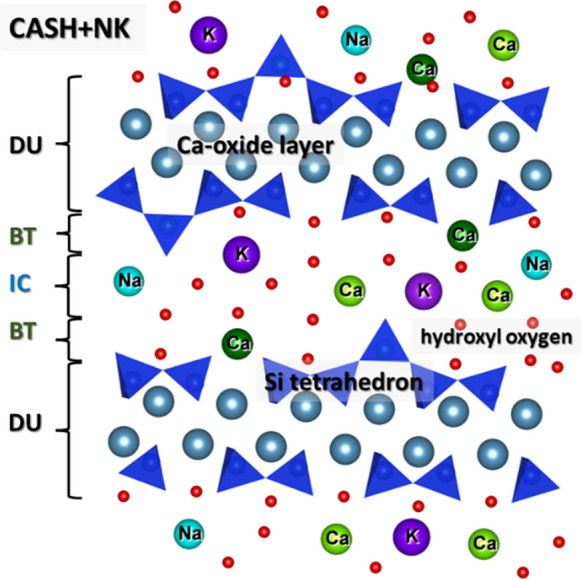
Schematic diagram for the CASH+NK model representing the C–S–H (defect tobermorite) structure. The Ca octahedral sheet is shown along with Si tetrahedra that form the dimeric unit (DU), the bridging tetrahedral site (BT), occupied with Si, Ca, or vacancy, and the interlayer cation site (IC), occupied with Ca, Na, K, or vacancy

Recently, a new C–S–H solid solution model, CASH+ , has been suggested by Kulik et al. [16]. The CASH+ model is a sublattice non ideal solid-solution model based on the defect tobermorite structure. It is constructed from end members and interaction parameters that account for possible substitutions of chemical moieties in the bridging tetrahedral (BT) and interlayer cation (IC) structural sites (Fig. 1). The model was then extended to account for the uptake of alkali metals and alkaline earth metals in Miron et al. [17] and work is being carried out to extend it for the uptake of Al and other elements. This CASH+NK model used in the present study is composed of several endmembers belonging to the core CASH+ model, plus those accounting for the uptake of Na and K (Table 1) with no account for the aluminum uptake. The endmembers are constructed by considering all possible combinations of moieties that can be substituted on the respective sublattice sites. In the case of alkali metals and alkaline earth metals, the endmembers are constructed assuming substitutions of moieties (e.g., KHOH^+^ and NaHOH^+^) in the IC sites only. By adjusting the stability of endmembers and the values of the interaction parameters between moieties on the respective sites, the model was tuned up against the experimental data on the uptake of cations, solubility, water content, and mean silicate chain length of C–S–H phases of various compositions. The model parameters were optimized against measured aqueous and solid phase compositions from experiments containing synthetic C–S–H in aqueous solutions at high water to solid ratios. However, this parameterization was never systematically tested against the pore solution data from hydrated cements with porosity normally around 10–15 vol%, where a rather small amount of pore water per unit mass of cement is available.

**Table 1:** Endmembers of CASH+NK model. Dimeric unit (DU), bridging tetrahedral (BT), interlayer cation (IC), and interlayer water (IW) are sites in the CASH+ solid solution model (Fig. 1) that can be occupied by different moieties (e.g., SiO_2_OH^−^ is a moiety in the BT site of the TSvh endmember)

End-members	Sites and sublattice formula moieties				Ca/Si	H/Si	Alkali/Si
	DU	BT	IC	IW			
T S v h	[Ca_2_Si_2_O_5_(OH)_2_]	SiO_2_OH^−^	H^+^	H_2_O	2/3	1	0
T S C h	[Ca_2_Si_2_O_5_(OH)_2_]	SiO_2_OH^−^	CaH(OH)_2_^+^	H_2_O	1	4/3	0
T v v h	[Ca_2_Si_2_O_5_(OH)_2_]	OH^−^	H^+^	H_2_O	1	3/2	0
T C v h	[Ca_2_Si_2_O_5_(OH)_2_]	CaOOH^−^	H^+^	H_2_O	3/2	3/2	0
T v C h	[Ca_2_Si_2_O_5_(OH)_2_]	OH^−^	CaH(OH)_2_^+^	H_2_O	3/2	2	0
T C C h	[Ca_2_Si_2_O_5_(OH)_2_]	CaOOH^−^	CaH(OH)_2_^+^	H_2_O	2	2	0
T S N h	[Ca_2_Si_2_O_5_(OH)_2_]	SiO_2_OH^−^	NaHOH^+^	H_2_O	2/3	7/6	1/3
T v N h	[Ca_2_Si_2_O_5_(OH)_2_]	OH^−^	NaHOH^+^	H_2_O	1	7/4	1/2
T C N h	[Ca_2_Si_2_O_5_(OH)_2_]	CaOOH^−^	NaHOH^+^	H_2_O	3/2	7/4	1/2
T S K h	[Ca_2_Si_2_O_5_(OH)_2_]	SiO_2_OH^−^	KHOH^+^	H_2_O	2/3	7/6	1/3
T v K h	[Ca_2_Si_2_O_5_(OH)_2_]	OH^−^	KHOH^+^	H_2_O	1	7/4	1/2
T C K h	[Ca_2_Si_2_O_5_(OH)_2_]	CaOOH^−^	KHOH^+^	H_2_O	3/2	7/4	1/2

The aim of the present paper is to assess the CASH+ model performance when modeling the alkali concentration in cement pore solutions. This was implemented by simulating the equilibrium pore solution composition of several completely hydrated Portland cements (PC) and PC blended with silica fume (SF) compositions compiled in Vollpracht et al. [15]. The total aqueous Na and K concentrations, calculated by the CASH+ model, were compared with the measured values, and it was recognized that the original CASH+ model overestimates the alkali metal binding at very low water to solid ratios and high Ca/Si. To obtain an improved agreement with the measured values, a further adjustment of the stability of the alkali metal containing CASH+ solid solution endmembers has been performed.

## Calculation setup.

The calculations were performed using the GEM-Selektor [18] v.3.9.3 geochemical modeling package and the thermodynamic data for cement phases from the Cemdata18 thermodynamic database [19], except for the model for C–S–H, where the CASH+ [16, 17] was used. The relevant stable phases and solid solutions considered in the calculations are given in Table 2. The thermodynamic data for the aqueous speciation, compatible with the Cemdata18 database, was taken from the PSI Nagra database GEMS version [20, 21].

**Table 2:** Phases that may appear in equilibrium calculations of hydrated PC and PC + silica fume mixtures. Unless otherwise specified, thermodynamic properties are taken from Lothenbach et al. [19]

Pure phases	Abbreviation	Solid solutions	Abbreviation
Portlandite	Por	CASH+ [16, 17]	C–S–H
Gypsum	Gp	M–S–H	M–S–H
Calcite	Cal	Monosulfate	Msf
Monocarbonate	Mca	Ettringite	Ett
Hemicarbonate	Hca	Straetlingite	Sra
Hydrotalcite	Htc	Hydrogarnet (Fe, Al, Si)	Hgt
Ferryhydrite	Fh		
Amorphous silica	Sam		
Brucite	Brc		

Some phases are modeled as ideal solid solutions, CASH+ as described in Kulik et al. [16]

Activity coefficients of aqueous species were calculated using the extended Debye-Hückel equation [22] and the common parameter values for KOH background electrolyte, ion size parameter *å* = *3.67* and *b*_*γ*_ = *0.123*. The non-hydrated cement recipes for PC, the silica fume composition, the clinker to silica fume ratios, and the liquid to solid ratios of many cements (supplementary information, Table SI1 and SI2) were taken from the cement pore solution dataset collected by Vollpracht et al. [15]. From the reported PC clinker and silica fume chemical analysis, the fractions given as loss on ignition (LOI), the amount of MnO, and the amount of TiO_2_ were summed up and considered as inert. The resulted inert part plus the oxide composition made of SiO_2_, CaO, Al_2_O_3_, Fe_2_O_3_, MgO, CO_2_, SO_3_, P_2_O_5_, Na_2_O, and K_2_O were recalculated to 100 g of clinker and 100 g of silica fume. The mixture compositions of clinker, silica fume and water of the reported ratios were given as input to the GEM-Selektor program. The equilibrium calculations result in a complete hydration of the reactive fraction (degree of reaction, reaction extent) prescribed in the input recipe.

The PC hydration calculation was done using the composition of a Portland cement blended with 4 mass% limestone according to Lothenbach et al. [23], as detailed in Table 3. The calculation for the hydration of a low pH cement (ESDRED) consisting of 40 mass% PC blended with 60 mass% SF was done using the compositions (see Table 3) reported in Lothenbach et al. [24]. The hydration of the clinker phases and the silica fume were calculated using the Parrot and Killoh empirical kinetics model [25] modified as described by Lothenbach et al. [23, 24].

**Table 3:** Composition of Portland cement blended with 4% limestone (PC4) from [23] and from PC used in the ESDRED cement from [24]

	PC4	PC(ESDRED)
*Normative phase composition [g/100 g]*^a^		
Alite	64.6	51.5
Belite	9.3	22.2
Aluminate	7.4	4.9
Ferrite	7.8	7.7
MgO(periclase)	0.9	1.0
CaO (free)	0.89	0.62
CaCO_3_	4.6^d^	3.0
Dolomite	–	1.5
CaSO_4_·2H_2_O	3.0^d^	1.9
CaSO_4_·0.5H_2_O		1.7
CaSO_4_		1.9
Syngenite	–	2.0
K_2_SO_4_^b^	1.3	0.19
Na_2_SO_4_^b^	0.20	0.2
*Present as solid solution in the clinker phases*		
K_2_O^c^	0.052	0.32
Na_2_O^c^	0.31	0.15
MgO^c^	0.87	0.50
SO_3_^c^	0.11	
Blaine surface area [m^2^/kg]	429	350

^a^From Rietveld analysis, expected errors: phases less than 10 wt.-%: ± 0.4%; phases 10–20 wt.-%: ± 1%: phases 30 wt.-% and more: ± 2%

^b^Readily soluble alkalis calculated from the concentrations of alkalis measured in the solution after 5 min agitation at a w/c of 10; present as alkali sulfates

^c^Calculated based on the chemical analysis and the mineralogical composition

The Parrot and Killoh model describes the rate $R$ of the hydration of the individual clinker phases as the minimum of the rates of the following processes:

nucleation and growth

$$R_{t} = \frac{{K_{1} }}{{ N_{1} }}\left( {1 - \alpha_{t} } \right)\left( { - \ln \left( {1 - \alpha_{t} } \right)} \right)^{{\left( {1 - N_{1} } \right)}} ,$$

diffusion

$$R_{t} = \frac{{K_{2} \left( {1 - \alpha_{t} } \right)^{2/3} }}{{1 - \left( {1 - \alpha_{t} } \right)^{1/3} }},$$

formation of hydration shell

$$R_{t} = K_{3} \left( {1 - \alpha_{t} } \right)^{{N_{3} }} .$$

The degree of hydration $\alpha$ at time $t$ (days) is expressed as

$$\alpha_{t} = \alpha_{t - 1} + \Delta t \cdot R_{t} - 1.$$

The rate can be adjusted for the influence of $\mathrm{w}/\mathrm{c}$ by multiplying it with

$$f\left( {{\text{w}}/{\text{c}}} \right) = \left( {1 + 3.333 \times \left( {{\text{H}} \times {\text{w}}/{\text{c}} - \alpha_{t} } \right)} \right)^{4} ,\;{\text{for}}\;\alpha_{t} > {\text{H}} \times {\text{W}}/{\text{C}}{.}$$

The values of parameters ${\mathrm{K}}_{1},{ \, \mathrm{N}}_{1},{ \, \mathrm{K}}_{2},{ \, \mathrm{K}}_{3}$ and ${\mathrm{N}}_{3}$ are given in Table 4.

**Table 4:** Parrot and Killoh model parameters used to calculate the hydration of the individual clinker phases as a function of time, from [24]

Parameter	Alite	Belite	Aluminate	Ferrite
K_1_	1.5	0.5	1.0	0.37
N_1_	0.7	1.0	0.85	0.7
K_2_	0.05	0.02	0.04	0.015
K_3_	1.1	0.7	1.0	0.4
N_3_	3.3	5.0	3.2	3.7
H	1.8	1.35	1.6	1.45

The amount of reacting silica fume (grams) as a function time $t$ (days) was calculated using the following empirical equation [24]:

$$SF = SF_{{{\text{inital}}}} - 100 \times \left( {0.1 + {\raise0.7ex\hbox{$5$} \!\mathord{\left/ {\vphantom {5 t}}\right.\kern-\nulldelimiterspace} \!\lower0.7ex\hbox{$t$}} + 16.67} \right)$$

At each time step, the kinetics models calculate the degree of reaction (reaction extent) for each clinker constituent and SF, which is a fraction of its initial mass allowed to equilibrate by GEM with the input amount of water to form possible hydrated cement phases (Table 2).

## Results

**Fig. 2:**
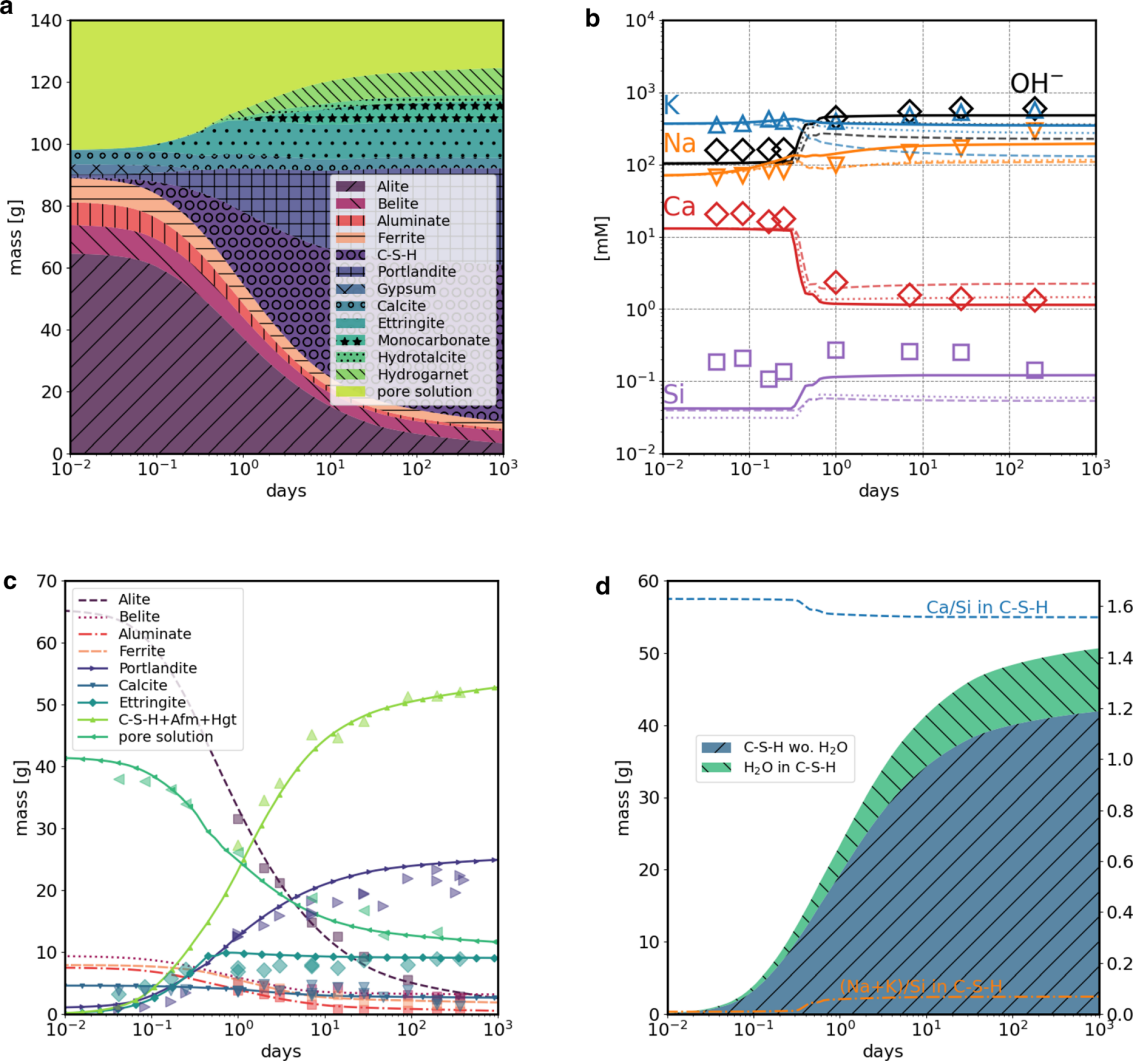
Hydration of Portland cement blended with 4% limestone according to Lothenbach et al. [23], at 1 bar, 20 °C. Calculations done using the fine-tuned CASH+ model. **a** evolution of masses of phases. **b** Calculated (lines) and measured (symbols) pore solution composition. Solid lines calculated with the fine-tuned CASH+ model, dashed lines with the original CASH+ model, dotted lines with the CSHQ model [19, 31]. **c** Amounts of clinker and hydrated products calculated and determined from XRD Rietveld analysis [23]. **d** calculated mass of C–S–H composed of H_2_O in C–S–H (structural + interlayer water) and “dry” part of C–S–H (no structural or interlayer water), Ca/Si and (Na+K)/Si in C–S–H [mol/mol] (right hand side y-axis)

The calculated pore solution concentrations in millimolarity (mM) units were compared with the reported measured values for the given cement mixture. To have a representative pore solution composition for the late stages of hydration, only the measurements done at > 19 days of hydration were considered in the comparison with the calculated values. The calculated and measured alkali pore solution concentrations change very little after this stage (Fig. 2b).

In order to better reproduce the measured Na and K pore solution concentrations, the CASH+ model was fine-tuned by weakening the stability of two endmembers containing K and Na in the interlayer site and Ca in the bridging tetrahedral site, "TCKh" and "TCNh", by adjusting their standard molar Gibbs energy with + 7.0 and + 5.0 kJ⋅mol^−1^, respectively (Table 5). The standard molar absolute entropy and standard molar heat capacity of the CASH+ endmembers are estimated from correlations with the molar volume [26] are within the estimation and measurement errors from the experimental values [16, 27]. The molar volumes were estimated so that the C–S–H density (*d*) experimental data are well reproduced (Fig. 6 in [16]). The assumption that endmembers with silica in the BT site (e.g., TSNh, TSKh, etc. with low Ca/Si) have a *d* = 2.5 g·cm^−3^ while all other endmembers (e.g., TCNh, TCKh, etc.) have a *d* = 2.7 g·cm^−3^ agrees with the C–S–H density values from low to high Ca/Si observed in measurements [28–30].

**Table 5:** Standard molar properties and uncertainties of fine-tuned alkali-metal CASH+ endmembers adjusted in this study. ${\delta G}_{298}^{\circ }$ is a 95% confidence interval (2σ)

Name	Formula	$\Delta {G}_{298}^{\circ }$	${G}_{298}^{\circ }$	${\delta G}_{298}^{\circ }$	${H}_{298}^{\circ }$	${S}_{298}^{\circ }$	${\delta S}_{298}^{\circ }$	${Cp}_{298}^{\circ }$	${\delta Cp}_{298}^{\circ }$	${V}_{298}^{\circ }$	${\delta V}_{298}^{\circ }$
	Sublattice CASH + endmembers	kJ·mol^−1^	kJ*·*mol^−1^	kJ·mol^−1^	kJ·mol^−1^	J mol^−1^·K^−1^	J·mol^−1^·K^−1^	J·mol﻿^−1^·K^−1^	J·mol﻿^−1^·K^−1^	cm^3^·mol^−1^	cm^3^·mol^−1^
	DU: BT: IC: IW										
TCNh	[(CaSiO_3_)_2_H_2_O]:{CaOOH}:{NaHOH}:{H_2_O}	5.0	− 4873.07	5.3	− 5297.14	377	38	356	107	142	6
TCKh	[(CaSiO_3_)_2_H_2_O]:{CaOOH}:{KHOH}:{H_2_O}	7.0	− 4891.65	5.3	− 5314.94	393	39	370	111	148	6
TCLih	[(CaSiO_3_)_2_H_2_O]:{CaOOH}:{LiHOH}:{H_2_O}	5.0	− 4903.79	6.4	− 5325.72	362	36	341	102	136	5
TCRbh	[(CaSiO_3_)_2_H_2_O]:{CaOOH}:{RbHOH}:{H_2_O}	7.0	− 4892.86	6.4	− 5306.34	438	44	412	124	165	7
TCCsh	[(CaSiO_3_)_2_H_2_O]:{CaOOH}:{CsHOH}:{H_2_O}	7.0	− 4900.86	6.4	− 5303.14	484	48	455	136	182	7

Sites: DU dimeric unit, BT bridging tetrahedral, IC interlayer cation, IW interlayer water. $\Delta {{\varvec{G}}}_{298}^{\circ }$ is the adjustment of endmember ${{\varvec{G}}}_{298}^{\circ }$ value relative to that given in [17]. The other endmembers of the CASH+NK model and its interaction parameters remain unchanged and are given in Kulik et al. and Miron et al. [16, 17]

Results of the simulated hydration of the Portland cement blended with 4% limestone [23] agree with the evolution of the phases amounts with time as determined by XRD Rietveld analysis (Fig. 2c). The sum of calculated amounts of C–S–H, monosulfate and siliceous hydrogarnet is comparable with the total amount of amorphous material determined from XRD. The calculation produces a typical late stage hydrated PC phases assemblage made from C–S–H, portlandite, ettringite, monocarbonate, hydrotalcite, hydrogarnet, and calcite added to the input clinker. The agreement with the measured pore water composition is improved when using the fine-tuned CASH+ , also when compared with the CSHQ model [19] (Fig. 2b). The measured Na, K, Ca, Si concentrations at the late stages of hydration (200 days) are: 300, 570, 1.3, 0.14 mM, in better agreement with the values predicted by the fine-tuned CASH+ model of 200, 350, 1.14, 0.12 mM when compared with 110, 130, 2.25, 0.05 mM and 117, 270, 1.5, 0.06 predicted by original CASH+ and CSHQ model, respectively. During the hydration, the calculated Ca/Si ratio in C–S–H decreases from 1.63 to 1.57, while the (Na + K)/Si ratio increases from 0.01 to 0.07 due to the increase of alkali concentrations with hydration time in the pore solution.

**Fig. 3:**
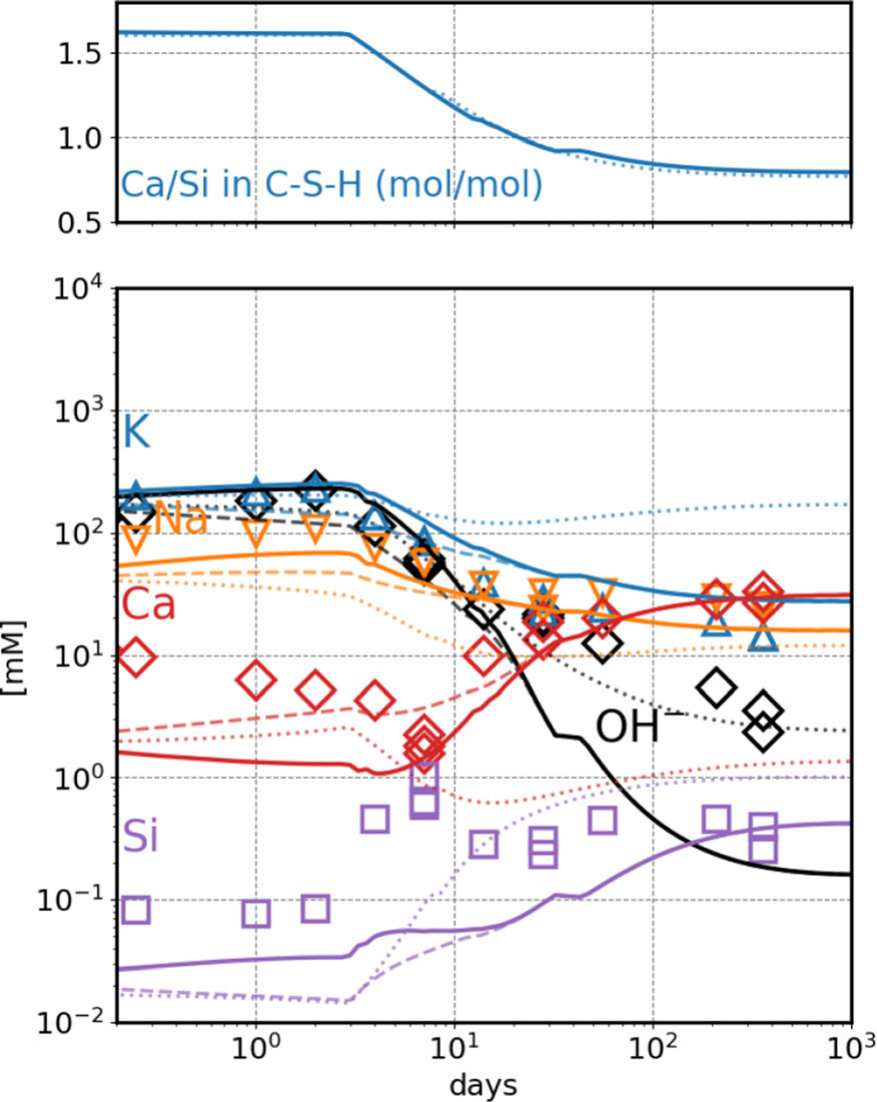
Hydration of Portland cement blended with silica fume according to Lothenbach et al. [24], 1 bar 20 °C. Solid lines: calculated using the fine-tuned CASH+ model. Dashed lines calculated with the original CASH+ model, dotted lines calculated with the CSHQ model [19, 31]

The calculated pore solution concentrations for the hydration of PC blended with silica fume as a function of time are compared with the measured values in Fig. 3. The measured concentrations of Na and K follow the calculated values, while the measured Ca, Si agree best at the later stages of hydration, which might be related to the slow rearrangement of C–S–H at early reaction times. The fine-tuned CASH+ model produces better agreements with the measured elemental concentrations (Na, K, Ca, Si) for the late stages of hydration (360 days) when compared with the CSHQ model except for OH^−^, which agrees better when using the CSHQ model. The calculated concentrations of 16, 28, 31, 0.4 mM using the CASH+ model are comparable to the 24, 14, 30, 0.33 mM values measured for Na, K, Ca and Si. Using the CSHQ model, the predicted concentrations for the same elements are 12, 170, 1.4, 1, respectively. When using the CASH+ model (original or fine-tuned), the measured OH- concentration (3 mM) is underestimated at the later hydration stages by around one order of magnitude (0.16 mM). The differences between the calculated values of the initial and the fine-tuned CASH+ model appear only in the early stages of hydration when the reaction material (clinker and silica fume) has a high Ca/Si ~ 9. This ratio decreases to 1 in the late stages where silica fume has reacted, leading to the formation of C–S–H with low Ca/Si ratio (Fig. 3). At these conditions, the TCNh and TCKh endmembers do not play a role in the CASH+ phase composition, and there is no significant effect due to the fine-tuning of the solid solution model.

**Fig. 4:**
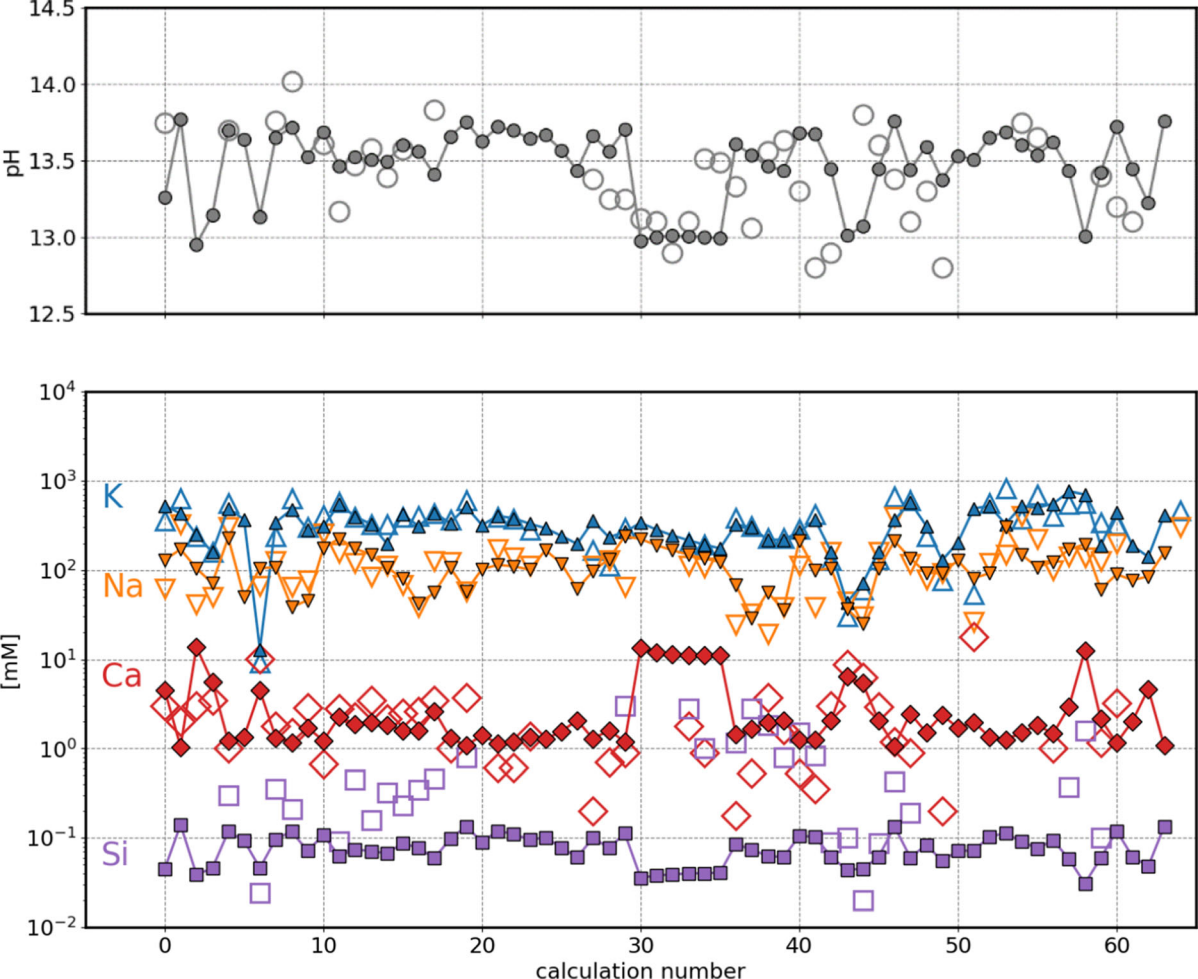
Comparison of measured (empty symbols) and calculated (full symbols) pore solution composition for hydrated PC compositions reported in Vollpracht et al. [15], 1 bar 20 °C

**Fig. 5:**
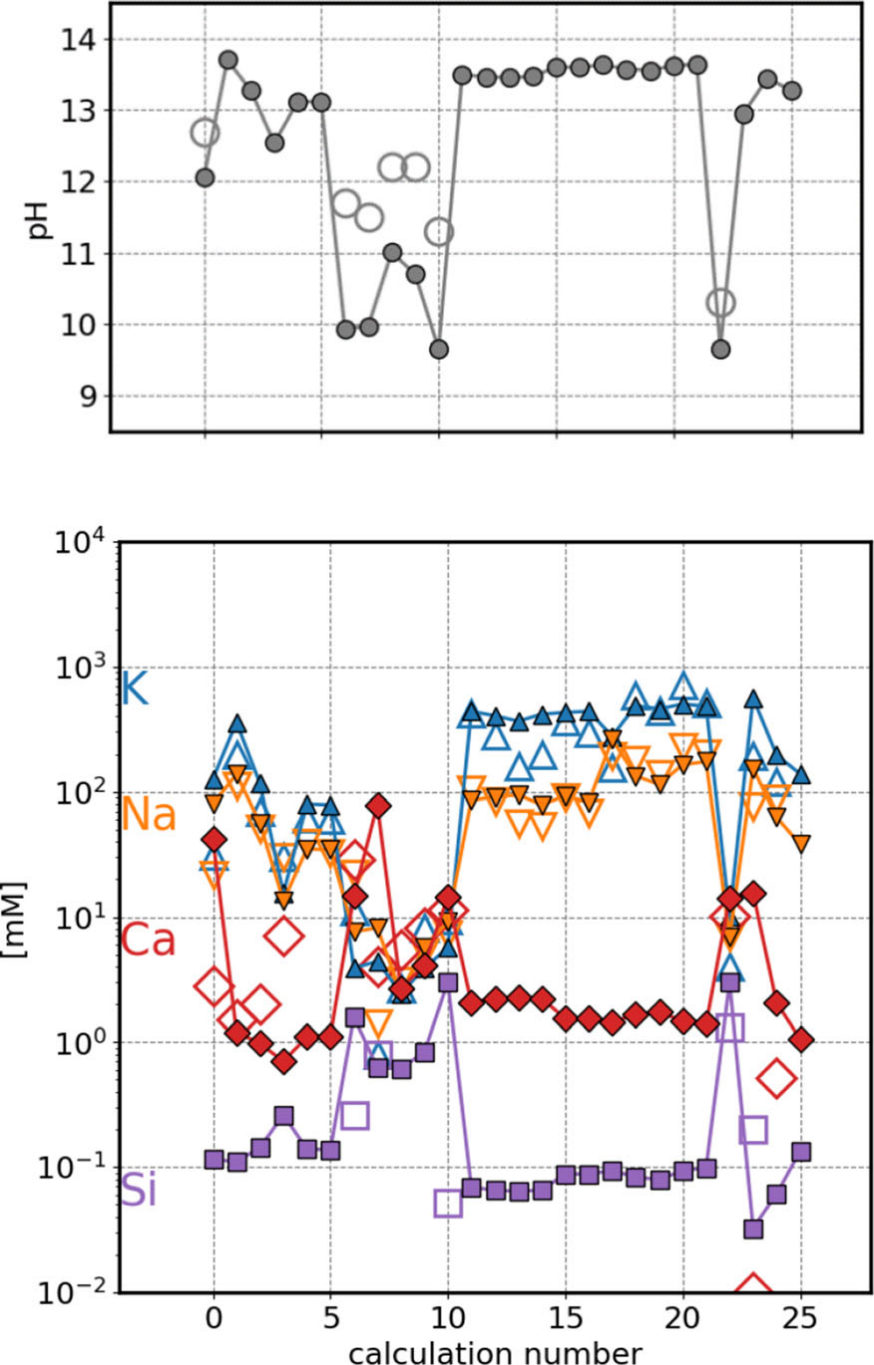
Comparison of measured (empty symbols) and calculated (full symbols) pore solution composition for hydrated PC blended with silica fume compositions reported in Vollpracht et al. [15], 1 bar 20 °C

In the next step, the pore water composition of 63 hydrated Portland cements (PC) and 26 PCs blended with silica fume, collected in Vollpracht et al. [15]*,* were calculated and compared with the measured values in Figs. 4 and 5. Additional information on the binder to water ratio and the stable phase assemblage for each calculation is given in the Supplementary information Table SI1 and Table SI2.

Using the fine-tuned CASH+ model, calculations predict pore solution compositions that reproduce the measured K and Na aqueous concentrations (Figs. 4 and 5). The measured Ca concentrations scatter more around the calculated values, while the measured Si concentrations have mostly larger values that the calculated ones. To note is that in many cases, the limit of detection for Si was ~ 0.04 mM [15], which is only slightly lower than most of the calculated values. The adjustment of "TCKh" and "TCNh" endmembers of the CASH+ model leads to a clearly better agreement between the measured and calculated K and Na concentrations in the pore solution of PC, while the improvements for the Ca and Si concentrations are less significant due the relative high scatter in the data (see Fig. SI1). Also, for the calculations of PC blended with silica fume, the adjustments to the stability of the two Ca-containing endmembers (TCKh and TCNh) further improved the accuracy of the alkali concentrations predictions, although the difference between the calculated and measured concentration was relatively low in both cases.

**Fig. 6:**
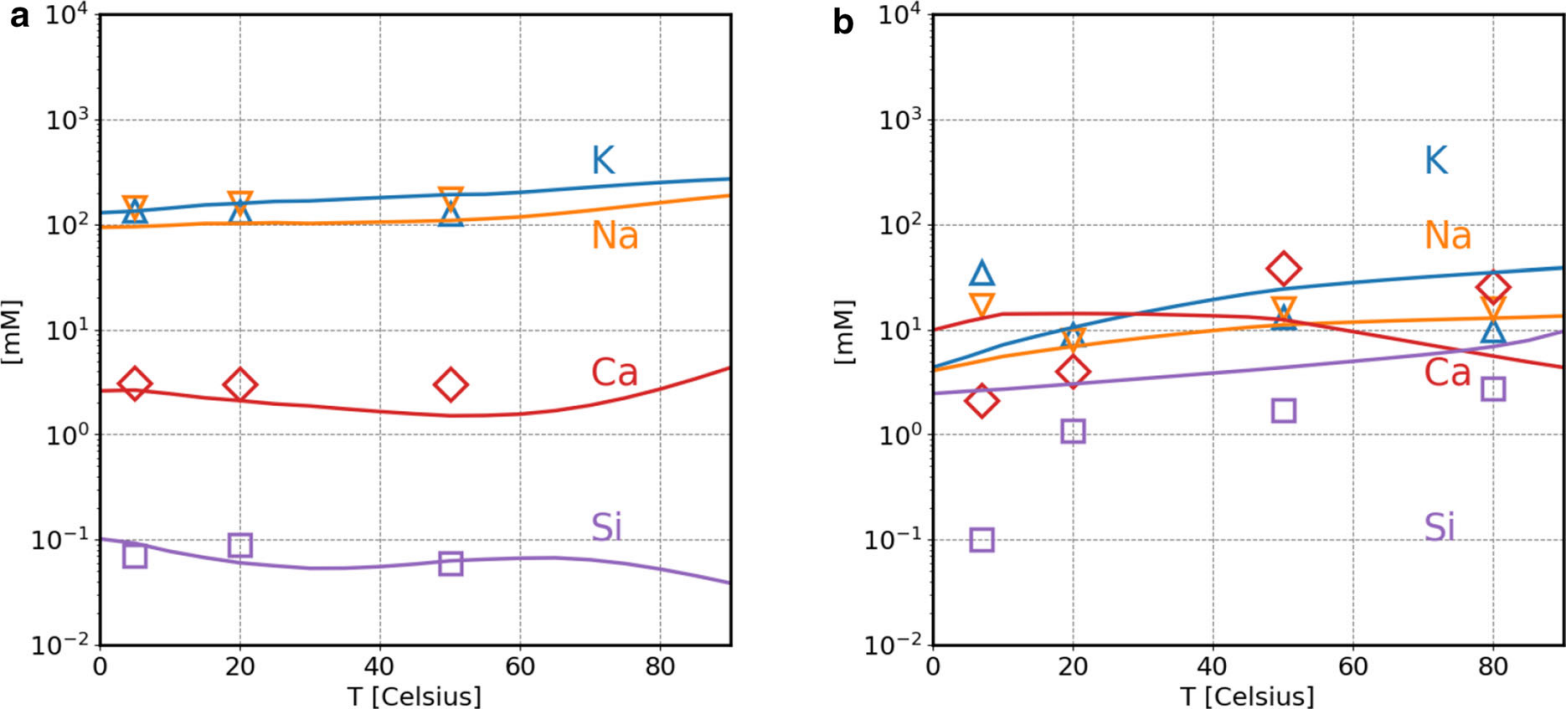
Comparison of measured (empty symbols) and calculated (curves) pore solution composition as a function of temperature. **a** PC [32], 150 days hydration time. **b** PC blended with silica fume after 56 days of hydration from Lothenbach 2013 (unpublished data) [15]

The temperature effect on the pore solution composition was simulated for PC and PC blended with silica fume SF (Fig. 6). Over the interval of 0–80 °C, little changes in the solution composition were predicted by the calculations, in agreement with the measured values. For the PC + SF systems, the K, Na, Ca, and Si concentrations are all predicted in the range of 40 and 140 mM with a larger scatter in the measured values (at 56 days of hydration), which is probably related to the strong effect of temperature on the silica fume reactivity [15]. The thermodynamic data in Cemdata18 database can be used up to of 100 °C [19]. The temperature dependence of the cement hydrate phases solubility is based on their values for the standard molar absolute entropy and standard molar heat capacity. These thermodynamic properties are derived from solubility experiments at different temperatures or from established estimation methods [19].

## Discussion

Early assessment of the CASH+ model when calculating the pore solution compositions of PC systems indicated that the model systematically underestimates the measured porewater K and Na concentrations (i.e., overestimates the uptake of K and Na in C–S–H at high Ca/Si) in PC and to a lower extent in blended cements. This, in turn, leads to underprediction of pH values, which can affect the calculated phase assemblage and the concentration of other elements in the pore solution.

The uptake of K and Na in C–S–H predicted by the initial CASH+ model [16, 17] is too strong, when applied to PC, where only little free pore solution is present. The original model was parameterized against several experimental datasets on the uptake of Na and K measured in dilute suspensions at different Ca/Si ratios and has shown a good agreement with the experimental data [17]. Nevertheless, the model endmembers with Ca in the BT sites, TCKh and TCNh, that determine the uptake of K and Na in systems with high Ca/Si ratios showed the lowest sensitivity to the experimental data as the alkali uptake is relatively low in C–S–H experiments with high liquid to solid ratio (> 20) leading to a large scatter in the measured alkali uptake, while for hydrated cements this ratio is << 1. The composite scaled sensitivities (effect of all observations on the parameter estimation) for the TCNh and TCKh of 140 and 70, are significantly smaller than the values for TSNh and TSKh of 800 and 400, which are parameters that are well constrained by the experiments.

All these factors influence the simulated chemical behavior of the systems. To get a better agreement with the measured K and Na concentrations in hydrated PC pore solutions, the ${G}_{298}^{\circ }$ of TCKh and TCNh endmembers were adjusted with + 7.0 and + 5.0 kJ⋅mol^−1^, respectively. The change is similar to or slightly larger than the estimated confidence intervals of ± 5.4 kJ⋅mol^−1^. The adjustments were obtained by trial and error, so that the least squares differences between the calculated and measured Na and K concentrations of completely hydrated PC pore solutions compiled in Vollpracht et al. [15] is minimized. This fine-tuning resulted in a weaker uptake of these alkalis in high Ca/Si systems such as PC. A much weaker underestimation of the K and Na concentrations by the original CASH+ model was observed for PC blended with silica fume systems. In these systems with high Si content and low Ca/Si, the uptake of K and Na is controlled by endmembers with Si and vacancy in the BT sites, TSKh, TvKh and TSNh, TvNh. These endmembers, especially the ones with Si in the BT site, were better constrained by the experimental data that were used to parameterize the model in Miron et al. [17]. This is shown in the model for the hydration of a PC blended with silica fume (Fig. 3), where a difference between the fine-tuned and the original CASH+ model is only seen for the initial stages of hydration when Ca/Si in C–S–H > 1. The disagreement with the measured OH^-^ is rather peculiar as it should be controlled by the C–S–H phase and mainly by the concentration of Na and K in the pore solution. Possible explanations for this could be the presence of other anions such as organic anions, chloride, fluoride or chromate, formate in the measured pore solution, from the used set accelerator and superplasticizer [24], which were not considered in the modelling, but can influence the hydroxide concentration at low molar concentrations. As discussed above, at these low Ca/Si ratio and low alkali concentration in solution (< 100 mM) the model reproduces the uptake of Na and K but also the measured pH for C–S–H solubility experiments in diluted experiments as shown in Miron et al. [17].In addition, alkali metals might also be bound by other phases e.g. by strätlingite as suggested by Winnefeld and Lothenbach [33]. Strätlingite formation is commonly observed and calculated in hydrated cements with Si-rich SCMs [34]. The fine-tuned CASH+ model predicts Na and K concentrations close to the measured values, and a change in the model predicted values for these elements will have an impact on the pH and the concentration of other elements like Ca and Si. This is visible in the calculations done using the CSHQ model that reproduce the measured pH at later stages of hydration but at the cost of a large overestimation of the K concentration. Thus, the CSHQ predicts much less K in C–S–H which in turn leads to larger calculated pH values that coincidentally agree with the measured ones.

**Fig. 7:**
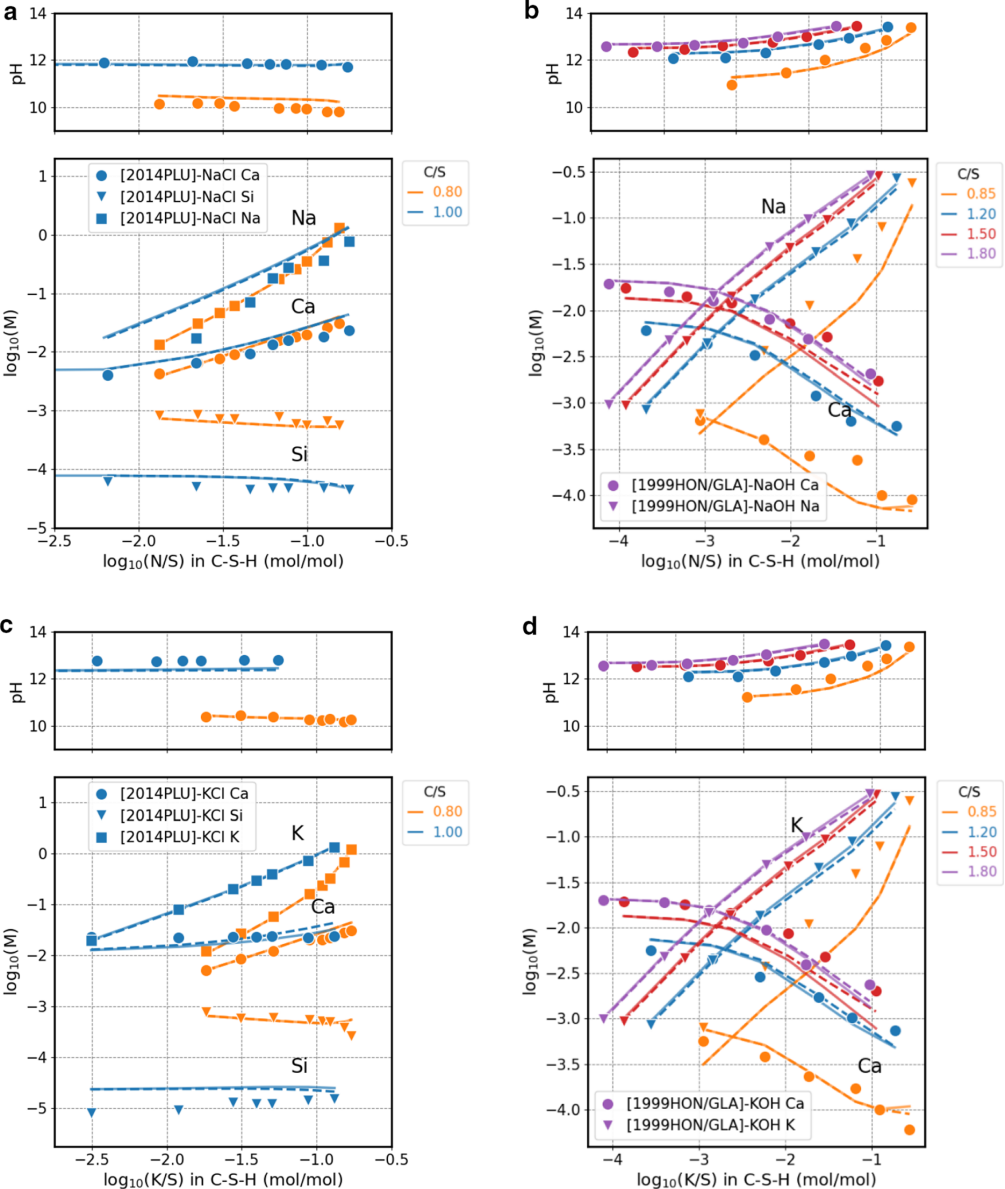
Comparison of measured (symbols) [12, 35] and calculated (lines) aqueous elemental concentrations for experiments on the uptake of Na in C–S–H used in Miron et al. [17]. Continuous curves calculated using the fine-tuned model, dashed line calculated with the original CASH+ model

Adjusting the stability of the TCKh and TCNh endmembers has an effect on calculations at high Ca/Si (> 1) and alkali concentrations > 10 mM in low liquid/solid systems (see Fig. 2b) and at > 100 mM in high liquid/solid systems (see Fig. 7b and d).

The low sensitivity of the adjusted parameters to the experiments in highly diluted experimental systems used in Miron et al. [17] to parameterize the original CASH+ model allowed to preserve the good agreement to the experimental data, as shown by the comparison of the measured data with the original CASH+ and the adjusted CASH+ model shown in Fig. 7. The fine-tuned CASH+ model predictions are now in agreement with both the experiments on the K and Na uptake used in Miron et al. [17] and better predicts the measured hydrated cements pore solution composition. For the uptake of other alkali metals like Li, Rb, Cs, the properties of their endmembers were estimated from those of Na and K assuming zero effect of the exchange reactions. Thus, the ${G}_{298}^{\circ }$ of TCLih was adjusted with + 5.0 kJ mol^−1^ and of TCRbh and TCCsh with + 7.0 kJ mol^−1^ (Table 5).

Additional uncertainties in the modeled K and Na pore solution concentration come from the errors in the measured alkali metals content of the clinker, the presence of additional anions such as organics, chloride, fluoride, or chromate not considered, the assumed uptake of alkali in C–S–H only, and the water content in C–S–H. For a completely hydrated PC with a water/ binder ratio of 0.4, the amount of interlayer water predicted by CASH+ model to be present in C–S–H is ~ 17 wt.% of C–S–H (9 g H_2_O per 100 g cement, Fig. 2d) being slightly more than half the weight of the pore solution (15.5 g pore solution per 100 g cement). Note that the gel water of C–S–H is considered as pore solution, while the interlayer water in C–S–H is considered as a part of the C–S–H and not of the pore solution. Therefore, the amount of interlayer water predicted by different C–S–H models will affect the calculated elemental concentrations in the pore solution.

To be able to fully describe the CASH+ system, the model needs to additionally consider the effect of Al. The incremental extension of the CASH+ model for the uptake of Al in the absence and presence of Na and K will be described in a future publication in preparation.

## Summary and conclusion

The CASH+ model was used in modeling calculations of hydrated PC and PC + silica fume SF mixtures. To get an improved agreement with the measured data the stability (G^o^_298_ value) of two CASH+ model endmembers, TCKh and TCNh, had to be adjusted with + 7.0 and + 5.0 kJ⋅mol^−1^, respectively. The agreement was maintained with the experiments used to originally parameterize the CASH+ model for the uptake of K and Na. The calculated K and Na concentrations predicted using the fine-tuned model are in good agreement with the measured values for both type of systems at different water to binder ratios, silica fume additions, and temperatures up to 90 °C. The fine-tuned CASH+ model is a significant improvement over the previous solid solution models (e.g. CSHQ [19]), and is therefore a better tool for predicting the cement pore solution composition.

## Supplementary Information

Below is the link to the electronic supplementary material.

Supplementary file1 (PDF 555 KB)

## References

[1] Alahrache S, Winnefeld F, Champenois JB (2016). Chemical activation of hybrid binders based on siliceous fly ash and Portland cement. Cem Concr Compos.

[2] Giron RGP, Chen X, La Plante EC (2018). Revealing how alkali cations affect the surface reactivity of stainless steel in alkaline aqueous environments. ACS Omega.

[3] Glasser FP (2013) Cements in Radioactive Waste Disposal (IAEA-TECDOC-CD--1701(Companion CD)). International Atomic Energy Agency (IAEA). https://inis.iaea.org/search/search.aspx?orig_q=RN:44122426

[4] Tits J, Wieland E (2018). Actinide sorption by cementitious materials. PSI Bericht Nr.

[5] Snellings R (2013). Solution-controlled dissolution of supplementary cementitious material glasses at pH 13: the effect of solution composition on glass dissolution rates. J Am Ceram Soc.

[6] Schöler A, Winnefeld F, Ben HM, Lothenbach B (2017). The effect of glass composition on the reactivity of synthetic glasses. J Am Ceram Soc.

[7] Thomas M (2011). The effect of supplementary cementing materials on alkali-silica reaction: a review. Cem Concr Res.

[8] Shi Z, Lothenbach B (2020). The combined effect of potassium, sodium and calcium on the formation of alkali-silica reaction products. Cem Concr Res.

[9] Taylor HFW (1997). Cement chemistry.

[10] Taylor HFW (1987). A method for predicting alkali ion concentrations in cement pore solutions. Adv Cem Res.

[11] Lothenbach B, Winnefeld F (2006). Thermodynamic modelling of the hydration of Portland cement. Cem Concr Res.

[12] Hong S-Y, Glasser FP (1999). Alkali binding in cement pastes: Part I. C-S-H Phase Cem Concr Res.

[13] L’Hôpital E, Lothenbach B, Scrivener K, Kulik DAA (2016). Alkali uptake in calcium alumina silicate hydrate (C-A-S-H). Cem Concr Res.

[14] Yan Y, Yang S-Y, Miron GD (2022). Effect of alkali hydroxide on calcium silicate hydrate (C-S-H). Cem Concr Res.

[15] Vollpracht A, Lothenbach B, Snellings R, Haufe J (2015). The pore solution of blended cements: a review. Mater Struct.

[16] Kulik DA, Miron GD, Lothenbach B (2022). A structurally-consistent CASH+ sublattice solid solution model for fully hydrated C-S-H phases: thermodynamic basis, methods, and Ca-Si-H2O core sub-model. Cem Concr Res.

[17] Miron GD, Kulik DA, Yan Y (2022). Extensions of CASH+ thermodynamic solid solution model for the uptake of alkali metals and alkaline earth metals in C-S-H. Cem Concr Res.

[18] Kulik DA, Wagner T, Dmytrieva SV (2013). GEM-Selektor geochemical modeling package: revised algorithm and GEMS3K numerical kernel for coupled simulation codes. Comput Geosci.

[19] Lothenbach B, Kulik DA, Matschei T (2019). Cemdata18: a chemical thermodynamic database for hydrated Portland cements and alkali-activated materials. Cem Concr Res.

[20] Thoenen T, Hummel W, Berner U, Curti E. (2014) The PSI/Nagra chemical thermodynamic database 12/07. PSI Bericht, Report No.: 14-04. Paul Scherrer Institut, Villigen, Switzerland

[21] Thoenen T, Kulik D (2003). Nagra/PSI chemical thermodynamic data base 01/01 for the GEM-Selektor (V.2-PSI) geochemical modeling code: release 28–02–03.

[22] Helgeson HC, Kirkham DH, Flowers GC (1981). Theoretical prediction of the thermodynamic behavior of aqueous electrolytes by high pressures and temperatures; IV, Calculation of activity coefficients, osmotic coefficients, and apparent molal and standard and relative partial molal properties to 600 degrees C and 5kb. Am J Sci.

[23] Lothenbach B, Le Saout G, Gallucci E, Scrivener K (2008). Influence of limestone on the hydration of Portland cements. Cem Concr Res.

[24] Lothenbach B, Rentsch D, Wieland E (2014). Hydration of a silica fume blended low-alkali shotcrete cement. Phys Chem Earth, Parts A/B/C.

[25] Parrot LJ, Killoh DC (1984). Prediction of cement hydration. Br Ceram Proc.

[26] Glasser L, Jenkins HDB (2016). Predictive thermodynamics for ionic solids and liquids. Phys Chem Chem Phys.

[27] Roosz C, Vieillard P, Blanc P (2018). Thermodynamic properties of C-S-H, C-A-S-H and M-S-H phases: results from direct measurements and predictive modelling. Appl Geochem.

[28] Muller ACA, Scrivener KL, Gajewicz AM, McDonald PJ (2013). Densification of C-S-H measured by 1H NMR relaxometry. J Phys Chem C.

[29] Muller ACA, Scrivener KL, Gajewicz AM, McDonald PJ (2013). Use of bench-top NMR to measure the density, composition and desorption isotherm of C-S-H in cement paste. Microporous Mesoporous Mater.

[30] Merlino S, Bonaccorsi E, Armbruster T (2001). The real structure of tobermorite 11Å: normal and anomalous forms, OD character and polytypic modifications. Eur J Mineral.

[31] Kulik DA (2011). Improving the structural consistency of C-S-H solid solution thermodynamic models. Cem Concr Res.

[32] Lothenbach B, Winnefeld F, Alder C (2007). Effect of temperature on the pore solution, microstructure and hydration products of Portland cement pastes. Cem Concr Res.

[33] Winnefeld F, Lothenbach B (2010). Hydration of calcium sulfoaluminate cements — Experimental findings and thermodynamic modelling. Cem Concr Res.

[34] Lothenbach B, Scrivener K, Hooton RD (2011). Supplementary cementitious materials. Cem Concr Res.

[35] Plusquellec G (2014) Analyse in situ de suspensions de silicate de calcium hydraté: application aux interactions ioniques à la surface des particules. Ph.D. Thesis

