# Supplementary material for: Porewater compositions of Portland cement with and without silica fume calculated using the fine-tuned CASH+NK solid solution model
Source: Mater Struct. 2022 Sep 24;55(8):212. doi: 10.1617/s11527-022-02045-0 (PMC9509308; doi:10.1617/s11527-022-02045-0)
Supplement: Supplementary file 1 — Supplementary file1 (PDF 555 KB) [file 11527_2022_2045_MOESM1_ESM.pdf]

Supplementary Information (SI)

Journal: Materials and Structures

Article Title: Porewater composition of ordinary Portland cement with and without silica fume calculated using the fine-tuned CASH+NK solid solution model

George Dan Miron[1,*], Dmitrii A. Kulik[1], Barbara Lothenbach[2]

[1] Laboratory for Waste Management LES, Paul Scherrer Institut, 5232 Villigen, Switzerland

[2] Concrete and Asphalt Laboratory, Empa, 8600 Dübendorf, Switzerland

*Corresponding author, dan.miron@psi.ch

Table SI1 *Predicted stable phases for the calculation of fully hydrated CEM-I type of cement recipes. water/binder (w/b) ratio.*

Ref.	#	w/b	C-S-H	Por	Cal	Ett	Msf	Mca	Hca	Htc	Hgrt	Brc
[1, 2]	0	0.50	+	+	+	+		+		+	+	
[3]	1	0.50	+	+		+				+	+	
[4]	2	0.60	+	+		+	+			+	+	
[4]	3	0.60	+	+		+				+	+	
[5]	4	0.50	+	+		+	+			+	+	
[6]	5	0.50	+	+		+	+			+	+	
[7]	6	0.50	+	+		+	+			+	+	
[7]	7	0.35	+	+		+				+	+	
[8]	8	0.50	+	+	+	+		+		+	+	
[9]	9	0.60	+	+	+	+				+	+	
[10]	10	0.40	+	+		+	+			+	+	
[9]	11	0.40	+	+	+	+				+	+	
[11]	12	0.50	+	+	+	+				+	+	
[9]	13	0.60	+	+	+	+				+	+	
[11]	14	0.50	+	+	+	+				+	+	
[11]	15	0.50	+	+	+	+				+	+	
[11]	16	0.50	+	+	+	+		+		+	+	
[9]	17	0.50	+	+	+	+				+	+	
[12]	18	0.45	+	+		+	+			+	+	
[13]	19	0.45	+	+	+	+		+		+	+	
[14]	20	0.40	+	+		+				+	+	
[15]	21	0.40	+	+		+	+			+	+	
[15]	22	0.45	+	+		+	+			+	+	
[15]	23	0.56	+	+		+	+			+	+	
[16]	24	0.50	+	+		+	+			+	+	
[16]	25	0.50	+	+		+	+			+	+	
[16]	26	0.50	+	+		+	+			+	+	
[17]	27	0.50	+	+		+	+			+	+	
[17]	28	0.50	+	+		+				+	+	
[18]	29	0.75	+	+		+	+			+	+	
[18]	30	0.50	+	+		+	+			+	+	
[18]	31	0.55	+	+		+	+			+	+	
[18]	32	0.60	+	+		+	+			+	+	
[18]	33	0.65	+	+		+	+			+	+	
[18]	34	0.70	+	+		+	+			+	+	
[18]	35	0.75	+	+		+	+			+	+	
[18]	36	0.75	+	+		+	+			+	+	
[18]	37	0.75	+	+		+	+			+	+	
[18]	38	0.75	+	+		+	+			+	+	
[18]	39	0.75	+	+		+	+			+	+	
[18]	40	0.75	+	+		+	+			+	+	
[18]	41	0.75	+	+		+				+	+	
[19]	42	0.40	+	+	+	+		+		+	+	
[19]	43	1.30	+	+	+	+		+		+	+	

[19]	44	1.30	+	+		+				+	+	
[20]	45	0.40	+	+	+	+		+		+	+	
[21]	46	0.40	+	+		+		+	+	+	+	
[22]	47	0.50	+	+	+	+		+		+	+	
[23]	48	0.40	+	+		+				+	+	
[24]	49	0.50	+	+		+	+			+	+	
[25]	50	0.50	+	+		+				+	+	
[26]	51	0.50	+	+		+	+		+	+	+	
[26]	52	0.50	+	+		+	+		+	+	+	
[26]	53	0.50	+	+	+	+		+		+	+	
[27]	54	0.50	+	+		+	+			+	+	
[28]	55	0.50	+	+		+	+			+	+	
[28]	56	0.50	+	+		+				+	+	
[28]	57	0.50	+	+		+				+	+	+
[29]	58	0.50	+	+		+	+			+	+	
[30]	59	0.50	+	+		+	+			+	+	
[31]	60	0.50	+	+		+				+	+	
[31]	61	0.36	+	+		+	+			+	+	
[31]	62	0.50	+	+		+				+	+	+
[32]	63	0.45	+	+		+	+			+	+	

For abbreviations of solid phase names, see Table 1 in the main text.

Table SI2 *Predicted stable phases for the calculation of fully hydrated CEM-I + silica fume (SF) mixture. Silica fume/clinker (sf/c), water/binder (w/b) ratio.*

Ref.	#	sf/c	w/b	C-S-H	Por	Gp	Cal	Ett	Msf	Hca	Stra	Htc	Hgt	Fh	M-S-H	Sam
[1, 2]	0	0.33	0.50	+			+	+			+	+	+			
[3]	1	0.11	0.50	+	+			+				+	+			
[3]	2	0.25	0.50	+				+				+	+			
[3]	3	0.43	0.50	+				+			+	+	+	+		
[15]	4	0.25	0.40	+				+			+	+	+			
[15]	5	0.25	0.45	+				+			+	+	+			
[33]	6	0.67	0.50	+		+	+	+			+			+	+	
[34]	7	0.67	0.55	+				+			+			+	+	
[24]	8	0.67	0.50	+				+			+	+		+		
[25]	9	0.67	0.50	+				+			+	+		+		
[25]	1	1.00	0.50	+		+					+			+	+	+
[26]	11	0.05	0.50	+	+			+	+	+		+	+			
[26]	12	0.11	0.50	+	+			+	+	+		+	+			
[26]	13	0.18	0.50	+	+			+	+	+		+	+			
[26]	14	0.11	0.50	+	+			+	+	+		+	+			
[26]	15	0.11	0.50	+	+			+	+	+		+	+			
[26]	16	0.11	0.50	+	+			+	+	+		+	+			
[26]	17	0.11	0.50	+	+		+	+				+	+			
[27]	18	0.05	0.50	+	+			+	+			+	+			
[27]	19	0.11	0.50	+	+			+	+			+	+			
[27]	20	0.05	0.50	+	+			+	+			+	+			

[27]	21	0.11	0.50	+	+			+	+		+	+			
[35]	22	1.00	1.00	+		+	+			+			+	+	+
[29]	23	0.11	0.50	+	+			+	+		+	+			
[30]	24	0.11	0.50	+	+			+	+		+	+			
[31]	25	0.25	0.35	+				+			+	+			

For abbreviations of solid phase names, see Table 1 in the main text.

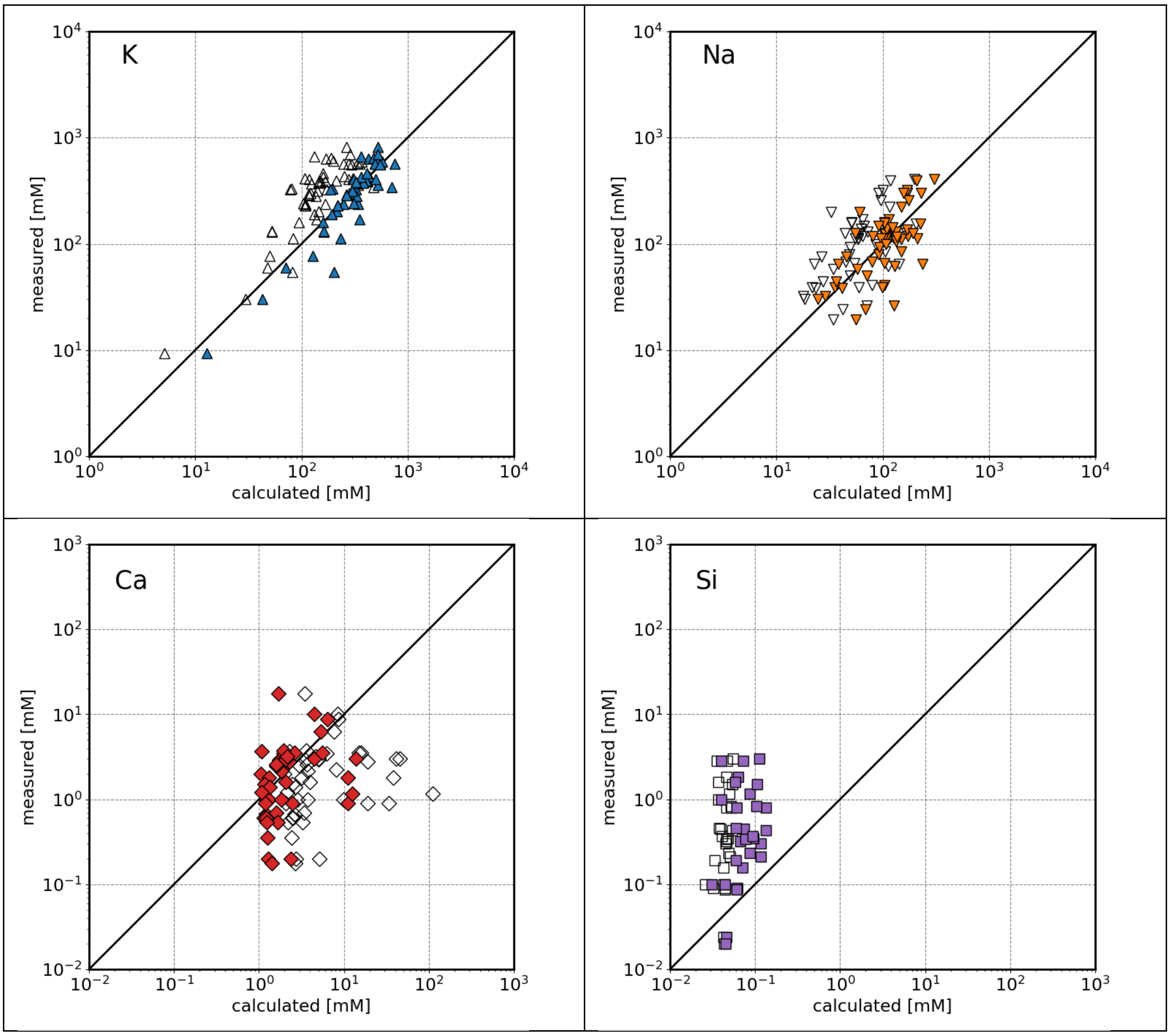

***Fig. SI1** Measured vs calculated pore solution composition of hydrated PC. Colored symbols are from calculations using the fine-tuned CASH+ model. Empty symbols are from calculations using the initial CASH+ model* [36].

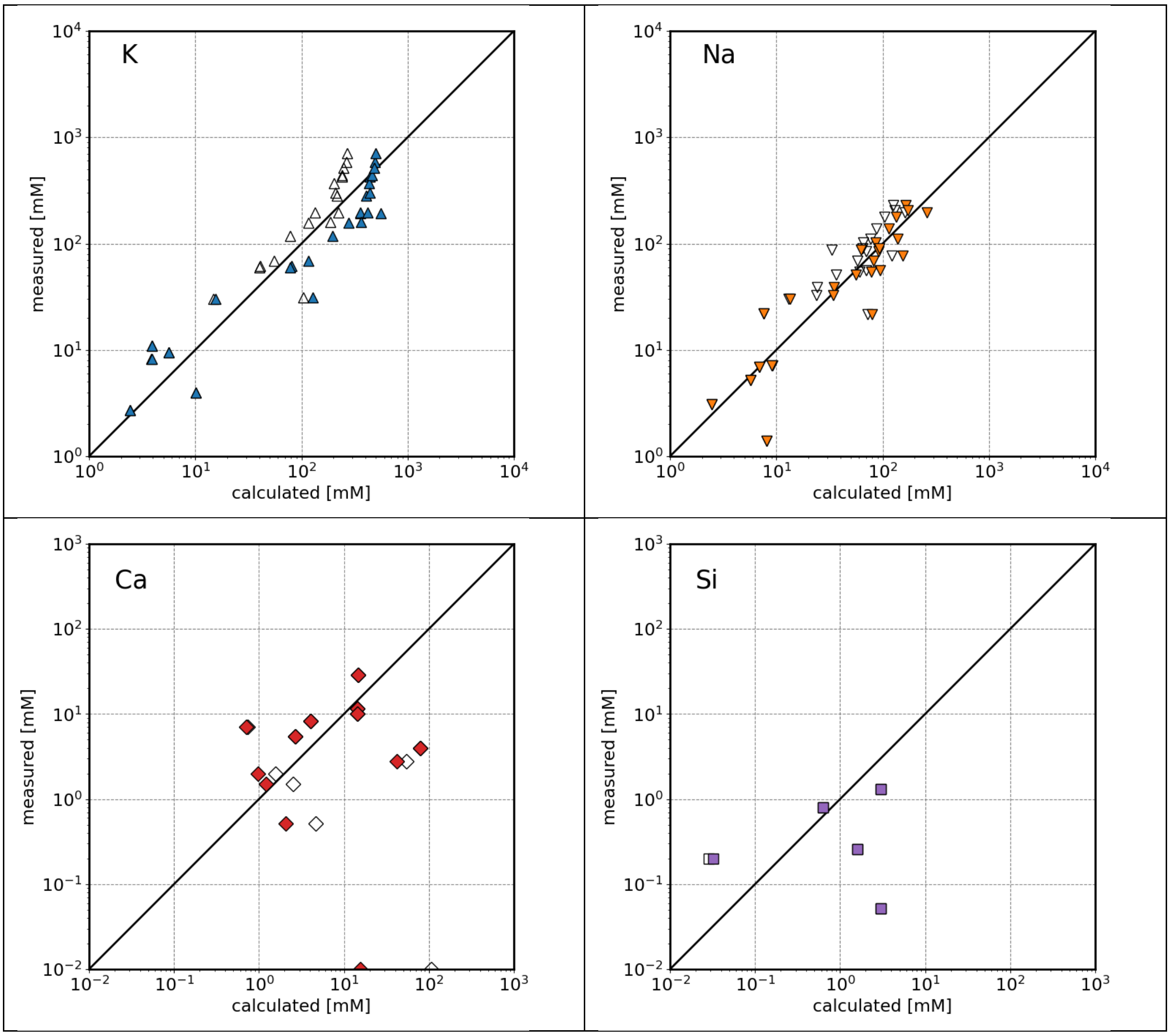

Fig. SI2 *Measured vs calculated pore solution composition of hydrated PC blended with silica fume. Colored symbols are from calculations using the adjusted CASH+ model. Empty symbols are from calculations using the initial CASH+ model* [36].

References

1. Brameshuber W VA (2007) Strukturuntersuchungen an 10 Jahre alten Zementsteinproben. Report No. F 928.
2. Schießl P, Meng B (1996) Grenzen der Anwendbarkeit von Puzzolanen im Beton. Report No. F 405.
3. Page CL, Vennesland O (1982) Pore solution composition and chloride binding capacity of silica fume-cement pastes. Report Number STF65 A82025. Trondheim
4. Schießl P, Härdtl R, Moersch J (1995) Untersuchungen zur Verwendung von Steinkohlenflugasche in Spannbeton mit sofortigem Verbund. Report No. F 430. Aachen
5. De Weerdt K, Haha M Ben, Le Saout G, et al (2011) Hydration mechanisms of ternary Portland cements containing limestone powder and fly ash. Cement and Concrete Research 41:279–291. https://doi.org/10.1016/J.CEMCONRES.2010.11.014

6. Brendle S, Rooij MR de, Breugel K van (2008) Pore Solution Evolution during the Early Portland Cement Hydration / Entwicklung der Porenlösung während der frühen Zementhydratation. Restoration of Buildings and Monuments 14:141–152. https://doi.org/10.1515/RBM-2008-6207

7. Rothstein D, Thomas JJ, Christensen BJ, Jennings HM (2002) Solubility behavior of Ca-, S-, Al-, and Si-bearing solid phases in Portland cement pore solutions as a function of hydration time. Cement and Concrete Research 32:1663–1671. https://doi.org/10.1016/S0008-8846(02)00855-4

8. Lothenbach B, Winnefeld F (2006) Thermodynamic modelling of the hydration of Portland cement. Cement and Concrete Research 36:209–226. https://doi.org/10.1016/j.cemconres.2005.03.001

9. Vollpracht A (2013) Pore solution of cement pastes with and without SCM.

10. Diamond S (1981) Effects of two Danish flyashes on alkali contents of pore solutions of cement-flyash pastes. Cement and Concrete Research 11:383–394. https://doi.org/10.1016/0008-8846(81)90110-1

11. Vollpracht A (2012) Einbindung von Schwermetallen in Portlandzementstein. Schriftenreihe Aachener Beiträge zur Bauforschung (18). RWTH Aachen University

12. Fraay A (1990) Fly Ash a Pozzolan in Concrete. Technical University Delft

13. Berry E, Hemmings R, Langley W, Carette G (1989) Beneficiated fly ash: Hydration, microstructure, and strength development in Portland cement systems. ACI SP-114. In: Malhotra V (ed) Fly Ash, Silica Fume, Slag, and Natural Pozzolans in Concrete. American Concrete Institute, Detroit, pp 241–273

14. Thomas JJ, Rothstein D, Jennings HM, Christensen BJ (2003) Effect of hydration temperature on the solubility behavior of Ca-, S-, Al-, and Si-bearing solid phases in Portland cement pastes. Cement and Concrete Research 33:2037–2047. https://doi.org/10.1016/S0008-8846(03)00224-2

15. Larbi JA, Fraay ALA, Bijen JMJM (1990) The chemistry of the pore fluid of silica fume-blended cement systems. Cement and Concrete Research 20:506–516. https://doi.org/10.1016/0008-8846(90)90095-F

16. Fujii K, Kondo W, Watanabe T (1970) Über die Hydratation von Portlandzement sofort nach dem Anmachen. Zement Kalk Gips 23:72–79

17. Silsbee M, Malek R, Roy D (1986) Composition of Pore Fluids Extruded from Slag-Cement Pastes. In: International Congress on the Chemistry of Cement, Rio de Janeiro. pp 263–269

18. Stassinopoulos E (1982) Untersuchung über die Zusammensetzung der flüssigen Phase und die Migrations- Prozesse in Zementpasten und Mörteln. Technical University Clausthal

19. Lothenbach B, Wieland E (2006) A thermodynamic approach to the hydration of sulphate-resisting Portland cement. Waste Manag 26:706–719. https://doi.org/10.1016/J.WASMAN.2006.01.023

20. Lothenbach B, Winnefeld F, Alder C, et al (2007) Effect of temperature on the pore solution, microstructure and hydration products of Portland cement pastes. Cement and Concrete Research 37:483–491. https://doi.org/10.1016/j.cemconres.2006.11.016

21. Lothenbach B, Le Saout G, Gallucci E, Scrivener K (2008) Influence of limestone on the hydration of Portland cements. Cement and Concrete Research 38:848–860. https://doi.org/10.1016/J.CEMCONRES.2008.01.002

22. Le Saoût G, Lothenbach B, Hori A, et al (2013) Hydration of Portland cement with additions of calcium sulfoaluminates. Cement and Concrete Research 43:81–94. https://doi.org/10.1016/J.CEMCONRES.2012.10.011

23. Lorenzo P, Goñi S, Hernández S, Guerrero A (1996) Effect of Fly Ashes with High Total Alkali Content on the Alkalinity of the Pore Solution of Hydrated Portland Cement Paste. Journal of the American Ceramic Society 79:470–474. https://doi.org/10.1111/J.1151-2916.1996.TB08146.X

24. Codina M, Cau-dit-Coumes C, Le Bescop P, et al (2008) Design and characterization of low-heat and low-alkalinity cements. Cement and Concrete Research 38:437–448. https://doi.org/10.1016/J.CEMCONRES.2007.12.002

25. García Calvo JL, Hidalgo A, Alonso C, Fernández Luco L (2010) Development of low-pH cementitious materials for HLRW repositories: Resistance against ground waters aggression. Cement and Concrete Research 40:1290–1297. https://doi.org/10.1016/J.CEMCONRES.2009.11.008

26. Schäfer D, Manconi A, Grandel S, Dahmke A (2005) Consequences of different kinetic approaches for simulation of microbial degradation on contaminant plume development. In: Reactive Transport in Soil and Groundwater: Processes and Models. Springer Berlin Heidelberg, pp 127–139

27. Duchesne J (2013) Alkali concentrations in pore solutions of pastes made of Portland cement with and without supplementary cementitious materials. Québec

28. Longuet P, Burglen L, Zelwer A (1973) La phase liquide du ciment hydrate. Revue des matériaux de construction 676:35–41

29. Chappex T (2012) The role of aluminium from supplementary cementitious materials in controlling alkali-silica reaction. École polytechnique fédérale de Lausanne

30. Hüttl R (2000) Der Wirkungsmechanismus von Steinkohlenflugasche als Betonzusatzstoff. Technical University Berlin

31. Andersson K, Allard B, Bengtsson M, Magnusson B (1989) Chemical composition of cement pore solutions. Cement and Concrete Research 19:327–332. https://doi.org/10.1016/0008-8846(89)90022-7

32. Nixon PJ, Bollinghaus R, Page CL, Canham I (2015) The effect of a Pfa with a high total alkali content on pore solution composition and alkali silica reaction. http://dx.doi.org/101680/macr19863813430 38:30–35. https://doi.org/10.1680/MACR.1986.38.134.30

33. Lothenbach B, Rentsch D, Wieland E (2014) Hydration of a silica fume blended low-alkali shotcrete cement. Physics and Chemistry of the Earth, Parts A/B/C 70–71:3–16. https://doi.org/10.1016/j.pce.2013.09.007

34. Bach TTH, Coumes CCD, Pochard I, et al (2012) Influence of temperature on the hydration products of low pH cements. Cement and Concrete Research 42:805–817. https://doi.org/10.1016/j.cemconres.2012.03.009

35. Lothenbach B (2013) Pore solution of cement pastes with high amounts of silica fume at different temperatures. Empa (unpublished).

36. Miron GD, Kulik DA, Yan Y, et al (2022) Extensions of CASH+ thermodynamic solid solution model for the uptake of alkali metals and alkaline earth metals in C-S-H. Cement and Concrete Research 152:106667. https://doi.org/10.1016/J.CEMCONRES.2021.106667
